# Combination therapy with an OX40L fusion protein and a vaccine targeting the transcription factor twist inhibits metastasis in a murine model of breast cancer

**DOI:** 10.18632/oncotarget.19967

**Published:** 2017-08-05

**Authors:** Anthony S. Malamas, Scott A. Hammond, Jeffrey Schlom, James W. Hodge

**Affiliations:** ^1^ Laboratory of Tumor Immunology and Biology, Center for Cancer Research, National Cancer Institute, National Institutes of Health, Bethesda, Maryland, USA; ^2^ MedImmune LLC, Gaithersburg, Maryland, USA

**Keywords:** OX40, cancer vaccines, combination immunotherapy, twist

## Abstract

OX40 is a costimulatory receptor that potentiates proliferation, survival, memory formation, and effector function of CD4^+^ and CD8^+^ T-cells, while overcoming the suppressive activity of regulatory T-cells (Tregs). Here, we explored the combination of an OX40L fusion protein (OX40L-FP) with a poxvirus-based cancer vaccine (MVA-Twist-TRICOM) to inhibit tumor metastasis in the 4T1 murine breast cancer model.

Contrary to the single agent treatments, the combination therapy significantly decreased the number of metastatic colonies per lung and prolonged survival. Depletion studies demonstrated that these effects were mediated by both CD4^+^ and CD8^+^ T-cells. The combination therapy a) increased the total number of T-cells in the CD4^+^Foxp3^-^ population and the CD4^+^ central and effector memory subsets within the lung, spleen, and draining lymph node, b) enhanced infiltration of CD4^+^ T-cells into metastatic areas of the lung, and (c) increased the number of functional CD8^+^ T-cells that produced IFNγ and TNFα. The combination therapy also promoted the development of KLRG1^-^CD127^+^ memory precursor CD8^+^ T-cells, while reducing those with a KLRG1^+^ terminally differentiated phenotype. Moreover, the combination of OX40L-FP and vaccine induced greater CD4^+^ and CD8^+^ Twist-specific responses. In addition, Tregs isolated from mice receiving the combination were also less immunosuppressive in *ex-vivo* proliferation assays than those from the OX40L-FP and MVA-Twist-TRICOM monotherapy groups. Such results provide the rationale to combine co-stimulatory agonists with cancer vaccines for the treatment of tumor metastasis.

## INTRODUCTION

OX40 (CD134) is a co-stimulatory receptor within the tumor necrosis factor receptor (TNFR) superfamily that is widely known to augment adaptive immune responses [1]. Its expression is transiently induced on CD4^+^ and CD8^+^ T-cells by TCR stimulation, and can peak anywhere from 24 hours to 4-5 days after antigen recognition. The ligand of OX40 (OX40L) is expressed on activated antigen-presenting cells (APCs), including B-cells, dendritic cells (DCs), and macrophages after CD40 ligation. OX40-OX40L interactions enhance the expansion, survival, memory formation, effector function, and recall responses of both CD4^+^ and CD8^+^ T-cells [2-7]. Furthermore, due to constitutive and inducible OX40 expression on mouse and human regulatory T-cells (Tregs), respectively, signals through this receptor can abrogate the suppressive activity of Tregs, prevent the induction of Tregs from effector T-cells, and reduce Foxp3 expression [8-12].

Targeting OX40 with exogenous agonists can significantly enhance anti-tumor immunity by overcoming self-tolerance and immunosuppressive mechanisms that contribute to tumor-induced T-cell anergy [3]. However, poorly immunogenic tumors are often weak responders to OX40 agonists due to limited numbers of tumor-infiltrating lymphocytes [13], thus requiring the need to combine such agents with other immune activating treatments. Cancer vaccines are attractive in combination with OX40 agonists based on their ability to overcome inadequate immune stimulation and increase the frequency of tumor-reactive T-cell populations. OX40 agonists may further enhance the proliferation and activity of antigen-specific T-cell populations generated upon vaccination to elicit robust anti-tumor immunity.

Here, we investigated this strategy in the poorly-immunogenic 4T1 murine tumor model of metastatic triple negative breast cancer (TNBC), combining a murine OX40L fusion protein agonist (OX40L-FP) with a poxvirus-based cancer vaccine (MVA-Twist-TRICOM). OX40L-FP is composed of 1) the extracellular receptor binding domain of murine OX40L, 2) isoleucine zipper trimerization domains derived from TNFR-associated factor 2, and 3) a murine IgG1 Fc domain that lacks effector function and facilitates Fcγ receptor clustering of the fusion protein following OX40 ligation [14-17]. The vaccine is composed of a Modified Vaccinia Ankara (MVA) virus incorporating transgenes for the tumor antigen Twist and a triad of costimulatory molecules (TRICOM), including B7-1, ICAM-1, and LFA-3. MVA-Twist-TRICOM converts infected cells into competent APCs that present Twist on the cell surface to induce antigen-specific CD4^+^ and CD8^+^ T-cell responses [18]. Twist is a transcription factor that mediates tumor metastasis, and its expression in the 4T1 tumor model is significantly greater in pulmonary metastases than in the primary tumor [19]. However, Twist is also considered to be a self-antigen due to its low expression in normal murine tissues of the lung, heart, muscle, and spleen [18], thus making it an ideal model antigen to study whether the combination therapy can break T-cell tolerance and amplify tumor antigen-specific immune responses that directly target metastatic lesions.

We show for the first time that combining OX40L-FP and vaccine enhances T-cell-mediated protection to significantly impair lung metastasis and prolong overall survival. The combination therapy increased CD4^+^ and CD8^+^ Twist-specific T-cell responses in both the periphery and lung, augmented CD4^+^ effector memory development, and biased the CD8^+^ effector memory population towards a memory precursor, rather than a terminally differentiated, phenotype. This therapy may have also reduced peripheral tolerance in part by abrogating the immunosuppressive effects of Tregs. Such results provide the rationale to combine co-stimulatory agonists with cancer vaccines for the treatment of tumor metastasis.

## RESULTS

### OX40L-FP enhances the proliferation of CD4^+^ T-cells, while overcoming the suppressive activity of Tregs *in vitro*

**Figure 1:**
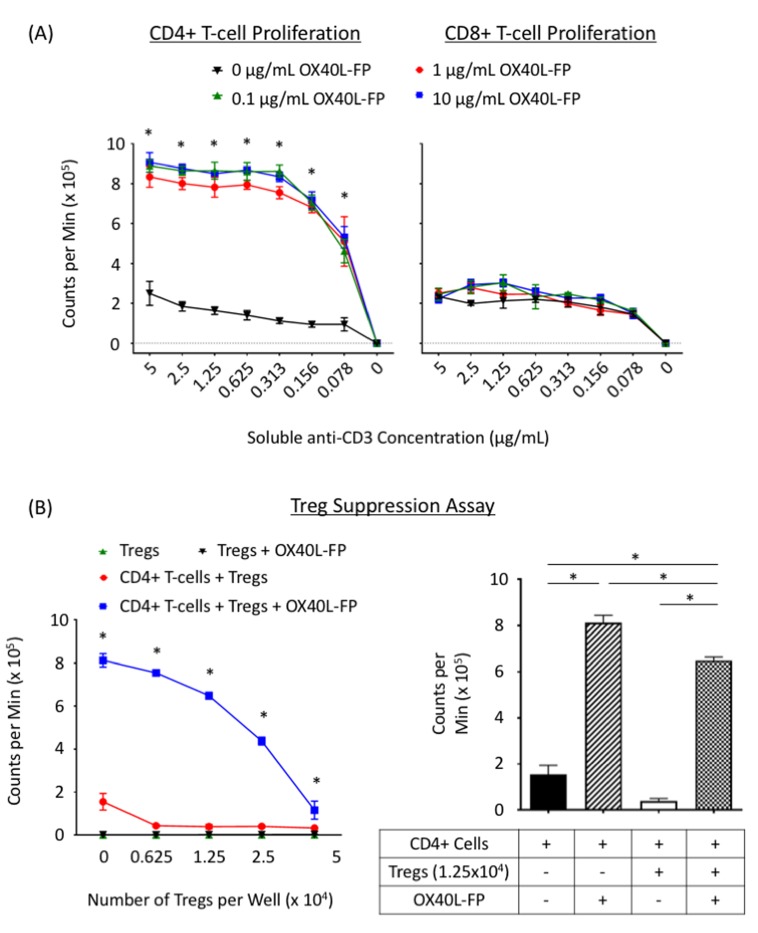
OX40L-FP enhances CD4^+^ T-cell proliferation and inhibits Treg suppression **A.**, *In-vitro* CD4^+^ and CD8^+^ T-cell proliferation assays. CD4^+^ and CD8^+^ T-cells isolated from WT Balb/c mice were co-cultured with irradiated APCs and various concentrations of soluble anti-CD3 and OX40L-FP. H^3^-thymidine uptake was measured after 4 days in culture. **B.**, Treg suppression assay. CD4^+^ responder cells from Balb/c mice were co-cultured with varying amounts of syngeneic Tregs, and stimulated with soluble anti-CD3, 10 µg/mL OX40L-FP, and irradiated APCs. H^3^-thymidine uptake was measured 3 days later.

The direct effects of OX40L-FP on T-cell proliferation were studied *in vitro* by culturing CD4^+^ and CD8^+^ T-cells from WT Balb/c mice, activated with soluble anti-CD3 mAb and APCs, in the presence or absence of OX40L-FP. Within 4 days, OX40L-FP induced a 3-fold increase in CD4^+^ T-cell proliferation at concentrations of 0.1, 1, and 10 µg/mL (*P* < 0.05) (Figure 1A). However, this agent did not directly affect the expansion of anti-CD3 activated CD8^+^ T-cells at any of the OX40L-FP concentrations tested.

The effects of OX40L-FP on Treg-mediated suppression *in vitro* were studied by evaluating their ability to inhibit the proliferation of an activated CD4^+^ T-cell responder population in response to the agonist. OX40L-FP had little to no effect on Treg proliferation, while it induced a 3-fold expansion of CD4^+^ responders stimulated with anti-CD3 mAb in the absence of Tregs. CD4^+^ proliferation gradually declined with increasing Treg numbers in the presence of OX40L-FP, although the CD4^+^ responder populations were still significantly greater than those cultured without OX40L-FP (Figure 1B), suggesting that the agonist was capable of overcoming the suppressive effects of Tregs.

### Combination OX40L-FP and vaccine expands both total and antigen-specific CD4^+^ and CD8^+^ T-cell populations in naive mice

**Figure 2:**
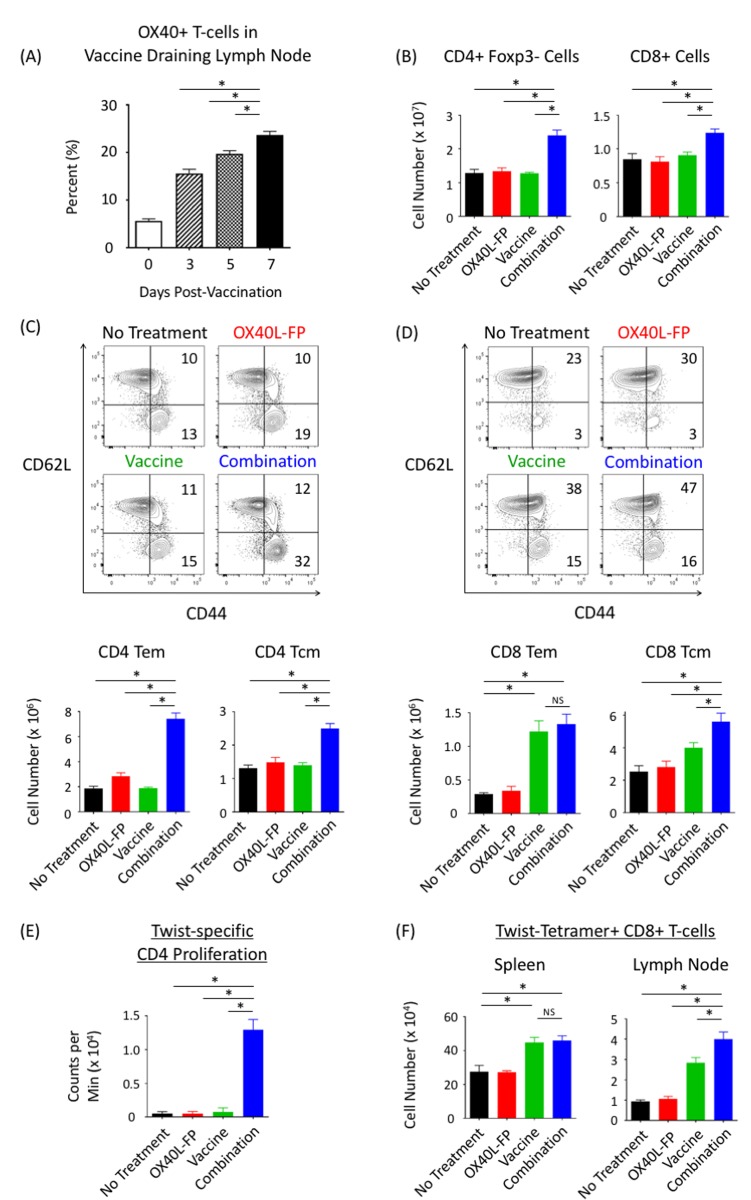
Combining OX40L-FP with MVA-Twist-TRICOM expands CD4^+^ and CD8^+^ T-cell populations in non‒tumor-bearing mice, while enhancing Twist-specific immune responses **A.**, Frequency of OX40^+^ T-cells within the activated CD3^+^CD4^+^Foxp3^-^CD44^+^ population of the DLN 3, 5, and 7 days after vaccination. **B.**, Absolute numbers of CD4^+^Foxp3^-^ and CD8^+^ T-cells in the spleens (*n* = 5) on day 21 post tumor transplant. Mice were given MVA-Twist-TRICOM on days 0, 7, and 14, and OX40L-FP on days 3, 6, 10, and 13 to maximize OX40 ligation on activated CD4^+^Foxp3^-^ T-cells. **C.**/**D.**, Representative contour plots (denoting frequencies) and absolute numbers of CD4^+^ and CD8^+^ Tem (CD44^+^CD62L^-^) and Tcm (CD44^+^CD62L^-^) populations. **E.**, Twist-specific CD4^+^ T-cell proliferation assay. Pooled CD4^+^ T-cells, isolated from spleens on day 28 post tumor transplant, were stimulated with MHC-II–restricted Twist peptide in the presence of irradiated APCs. H^3^-thymidine uptake was measured 4 days later. **F**. Absolute numbers of Twist-tetramer^+^ CD8^+^ T-cells in the spleens and DLNs.

We next explored how OX40L-FP and MVA-Twist-TRICOM vaccine can enhance total and antigen-specific CD4^+^ and CD8^+^ T-cell responses in non-tumor-bearing Balb/c mice. To determine an appropriate dosing schedule, the expression kinetics of OX40 receptor on CD4^+^Foxp3^-^ T-cells in the vaccine draining lymph node (DLN) were examined after a single dose of MVA-Twist-TRICOM. Prior to vaccination, 5% of activated (CD44^+^) CD4^+^Foxp3^-^ T-cells expressed OX40, and the frequency of this population then significantly rose to 15%, 20%, and 23% on days 3, 5, and 7 post-vaccination, respectively (Figure 2A). In order to ensure ligation of OX40L-FP with its receptor on activated T-cells upon vaccination, we decided to administer OX40L-FP both 3 and 6 days after the prime and boost vaccinations in our combination treatment regimen. Therefore, MVA-Twist-TRICOM was administered on days 0, 7, and 14 in naïve Balb/c mice, while OX40L-FP was given on days 3, 6, 10, and 13. By day 21, the total number of CD4^+^ and CD8^+^ T-cells in the spleen were 1.8-fold and 1.4-fold greater, respectively, in the combination group than in the untreated and monotherapy controls (*P* < 0.05) (Figure 2B). We also discovered that the combination therapy significantly increased the CD4 effector memory (Tem; CD44^+^CD62L^-^) and central memory (Tcm; CD44^+^CD62L^+^) T-cell populations in the spleen (Figure 2C). Unlike the Tem population, the expansion of CD4^+^ Tcm cells was primarily mediated by an overall increase in the entire CD4^+^Foxp3^-^ parent population, as the combination therapy had minimal effects on Tcm frequency. A similar trend was observed in the CD8^+^ Tcm population. However, the combination therapy did not increase the size of the CD8^+^ Tem population. Vaccine alone induced a significant 4-fold increase in CD8^+^ Tem numbers, but co-administration of OX40L-FP did not further expand this population (Figure 2D).

In addition to expanding the total polyclonal CD4^+^ and CD8^+^ T-cell populations, the combination therapy also enhanced Twist-specific responses. On day 21, CD4^+^ T-cells isolated from splenocytes in the combination group proliferated 10 times more rapidly than those from the untreated and monotherapy control groups upon re-stimulation with an MHC-II‒restricted Twist peptide (Figure 2E). MHC-I‒restricted Twist-tetramer staining demonstrated that while the total number of Twist-specific CD8^+^ T-cells increased following vaccination, the addition of OX40L-FP did not further expand this population in the spleen. However, the combination therapy increased the Twist-specific CD8^+^ T-cell population in the vaccine DLN compared to the controls (Figure 2F).

### Combination therapy reduces lung metastasis and prolongs survival in the 4T1 breast cancer tumor model in a CD4^+^ and CD8^+^ T-cell dependent manner

**Figure 3:**
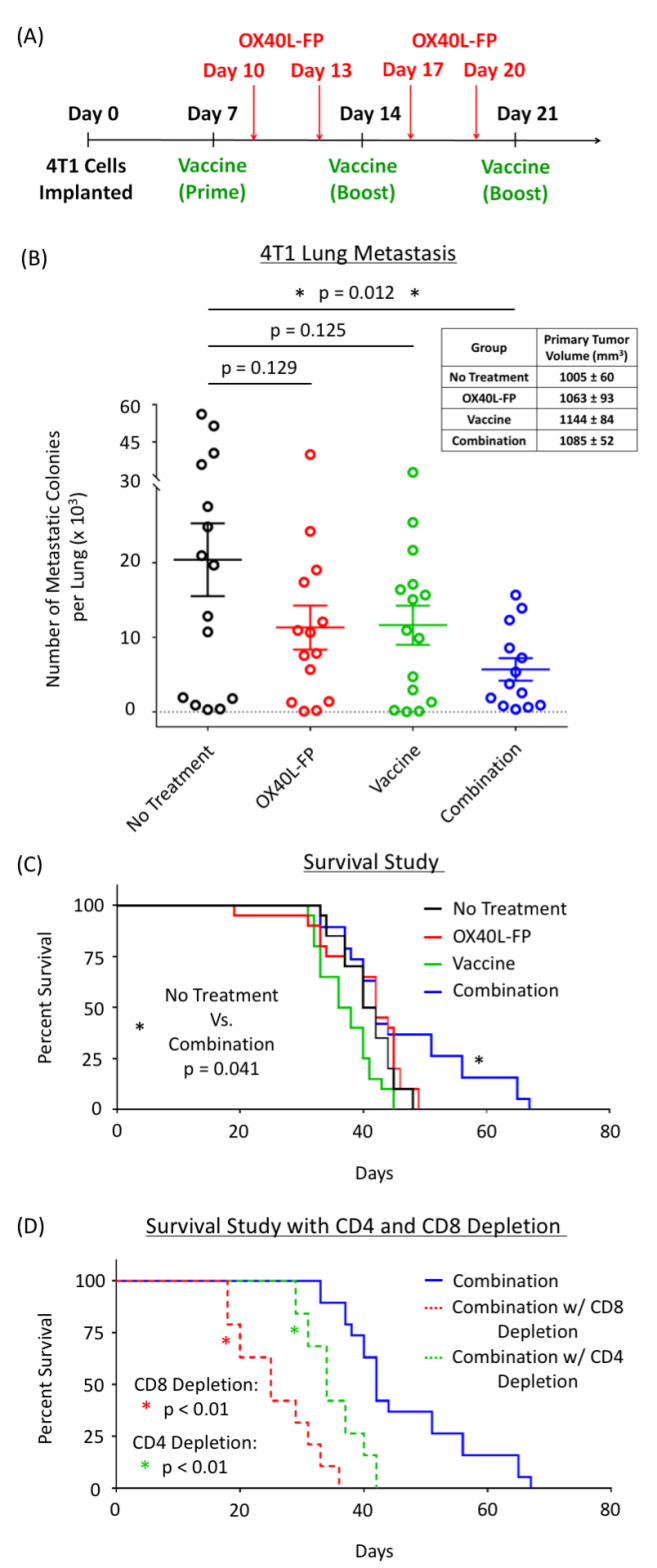
Combination of OX40L-FP and MVA-Twist-TRICOM reduces lung metastasis and prolongs survival in 4T1 murine breast tumor model in a CD4^+^ and CD8^+^ T-cell dependent manner **A.**, Diagram of the treatment schedule. **B.**, Number of clonogenic 4T1 colonies cultured from the lungs (*n* = 15) of 4T1 tumor-bearing mice on day 28 post tumor transplant. Inset panel: primary tumor volumes on day 28. **C.**, Survival curves from a 4T1 study where primary tumors were surgically resected on day 15. **D.**, Survival curves in response to CD4^+^ and CD8^+^ depletion.

After establishing that the combination of OX40L-FP and MVA-Twist-TRICOM enhances the immunomodulatory effects achieved by each agent alone, our goal was to exploit this strategy for the treatment and prevention of lung metastasis in the 4T1 tumor model of TNBC. The treatment schedule utilized for these studies is presented in Figure 3A. As single agents, OX40L-FP and MVA-Twist-TRICOM had no effect on the formation of lung metastases in 4T1 tumor-bearing mice. Metastasis was impaired only when the two treatments were combined together, as demonstrated by a significant reduction in the number of clonogenic metastatic colonies that were cultured *ex vivo* after lung harvest (Figure 3B). No effect on primary tumor growth was observed (Figure 3B, inset), possibly due to its low Twist expression [19]. The significant reduction in lung metastasis by the combination therapy led to a corresponding increase in survival compared to the controls (Figure 3C). Depleting CD4^+^ and CD8^+^ T-cells completely abrogated its effects on overall survival (Figure 3D).

### Combination therapy expands the total CD4^+^ Foxp3^-^ T-cell population, and enhances CD4^+^ Twist-specific responses at the metastatic tumor site

**Figure 4:**
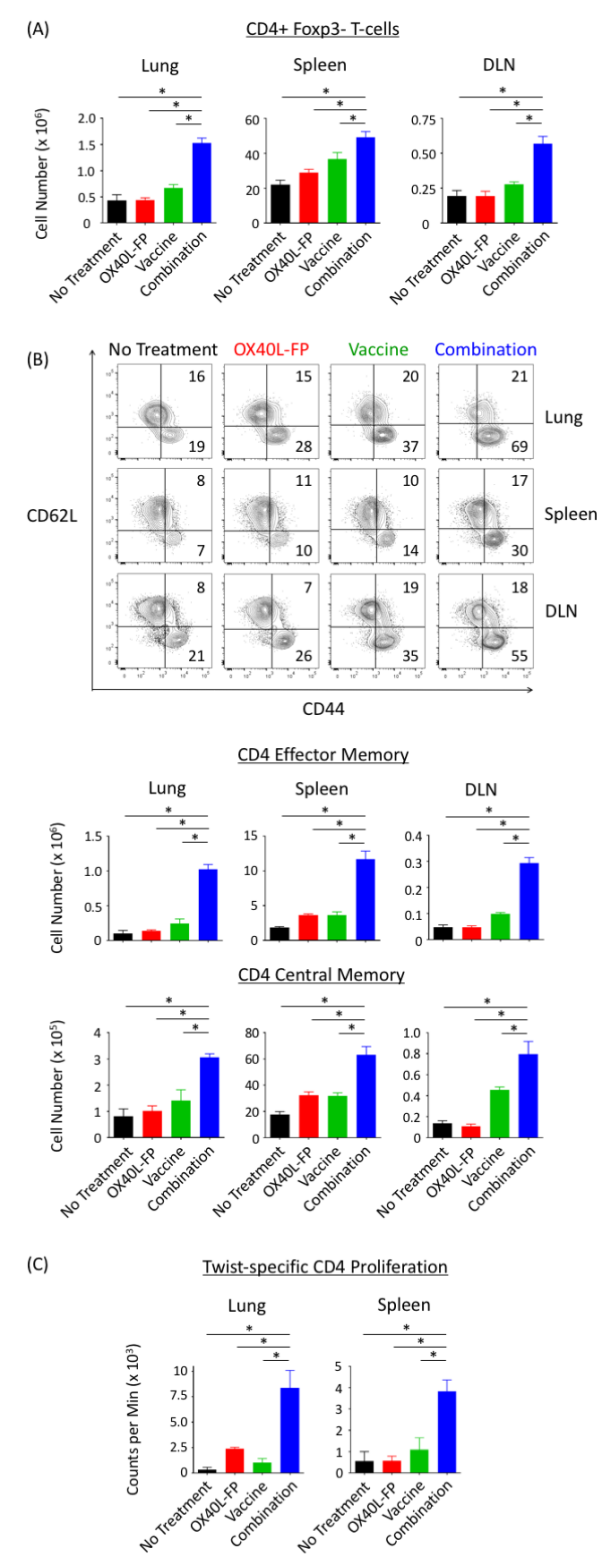
Combination therapy augments the total CD4^+^Foxp3- population, and CD4 Tem and Tcm subsets, while enhancing Twist-specific T-cell responses in 4T1 tumor-bearing mice **A.**, Absolute number of CD4^+^Foxp3^-^ T-cells in the lungs, spleens, and DLN (*n* = 5) on day 28 post tumor transplant. **B.**, Representative contour plots (denoting frequencies) and absolute numbers of CD4^+^ Tem (CD44^+^CD62L^-^) and Tcm (CD44^+^CD62L^+^) populations. Events were pre-gated on CD3^+^CD4^+^Foxp3^-^ T-cells. **C**., Twist-specific CD4^+^ T-cell proliferation assay. CD4^+^ T-cells pooled from lungs and spleens on day 28 post tumor transplant were stimulated with MHC-II–restricted Twist peptide in the presence of irradiated APCs. H^3^-thymidine uptake was measured 4 days later.

The combination of OX40L-FP and MVA-Twist-TRICOM significantly expanded the total number of CD4^+^Foxp3^-^ T-cells at the metastatic tumor site (lung) by at least 2.1-fold compared to the individual treatments 28 days after tumor transplant (Figure 4A). The combination therapy also increased the frequency of the CD4^+^ Tem subpopulation (Supplementary Figure 1), resulting in over a 4-fold enrichment in absolute cell number compared to the monotherapy controls (Figure 4B). Total numbers of CD4^+^ Tcm cells were also statistically the greatest in the combination group (Figure 4B), although no frequency changes were observed (Supplementary Figure 1). Similar combination effects were observed in the total CD4^+^Foxp3^-^ population, and the CD4^+^ Tem and Tcm subsets, of the periphery (spleen and vaccine DLN) (Figure 4A and 4B).

Co-administration of OX40L-FP and MVA-Twist-TRICOM also generated greater CD4^+^ Twist-specific responses. CD4^+^ T-cells isolated from lungs and spleens of mice that received the combination therapy significantly expanded more rapidly than those isolated from the individual groups following *ex-vivo* stimulation with an MHC-II‒restricted Twist peptide (Figure 4C). This result suggests that the combination therapy not only expanded the entire polyclonal CD4^+^Foxp3^-^ T-cell population in the metastatic tumor site and periphery, but also the subpopulation specific for the tumor antigen Twist.

**Figure 5:**
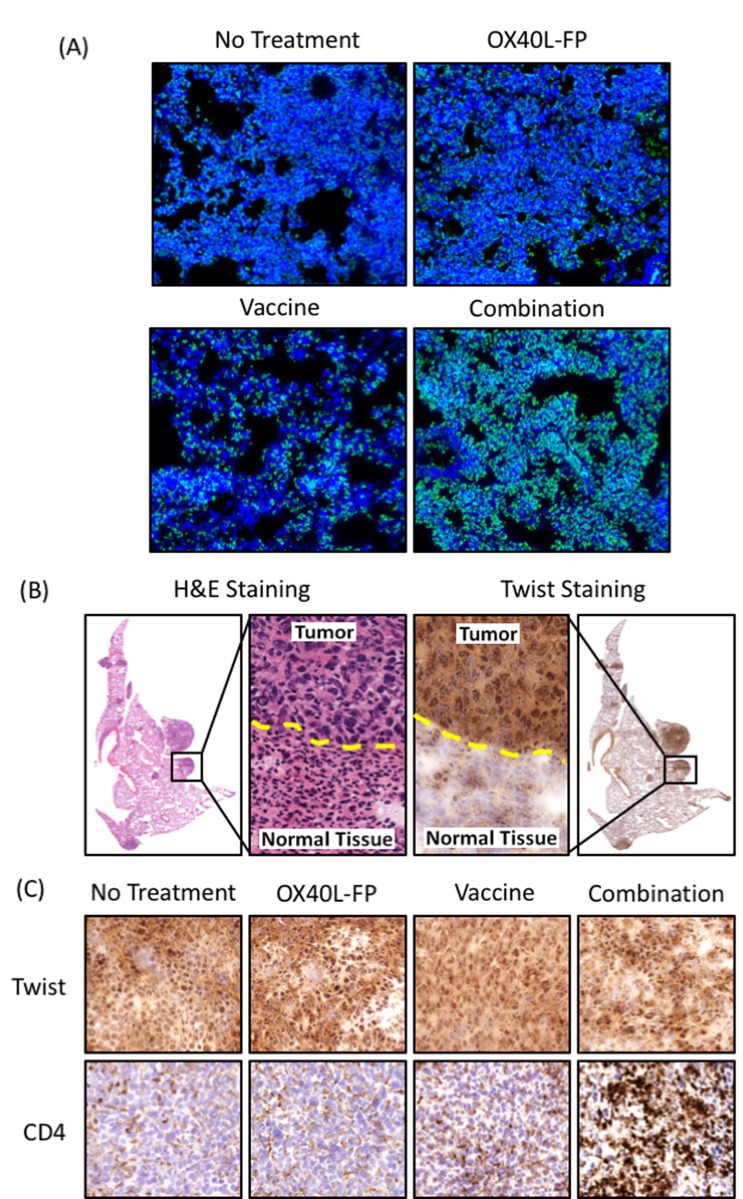
Combination therapy increases CD4^+^ T-cell infiltration into 4T1 metastatic sites of the lung **A.**, Representative immunofluorescence images of CD4 (green) and nuclear (blue) staining at 10x magnification. **B.**, H&E and Twist stains demonstrating co-localization of Twist expression with regions of 4T1 metastasis in the lung. **C.**, Representative images of CD4 staining (bottom panels) in areas of the lung positive for Twist (top panels).

Immunofluorescence imaging verified that the combination therapy markedly enhanced CD4^+^Foxp3^-^ immune infiltrate into the metastatic lung tissue compared to the individual therapies (Figure 5A). We separately stained lung sections for Twist expression to determine whether the combination therapy also led to greater co-localization of CD4^+^ T-cells with micro- and macro-metastatic deposits of 4T1 tumor cells. There were no differences in infiltration of CD8^+^ T-cells (not shown). Areas of elevated Twist expression enabled us to selectively identify metastatic 4T1 cells, as these areas specifically corresponded with H&E staining characteristic of tumor tissue (Figure 5B). Immunohistochemistry (IHC) analysis of Twist and CD4^+^ expression revealed that the combination therapy strikingly enhanced T-cell infiltration into 4T1 metastatic lesions of the lung compared to the OX40L-FP and MVA-Twist-TRICOM monotherapies (Figure 5C). Finally, we interpret the reduced signal for Twist in mice treated with combination therapy not as reduced Twist expression, but as fewer Twist expressing cells. On high power examination, there is no quantitative difference in Twist expression on a per cell basis.

### Combination therapy increases the frequency of functional CD8^+^ cells and the total number of Twist-specific CD8^+^ T-cells in the 4T1 tumor model

**Figure 6:**
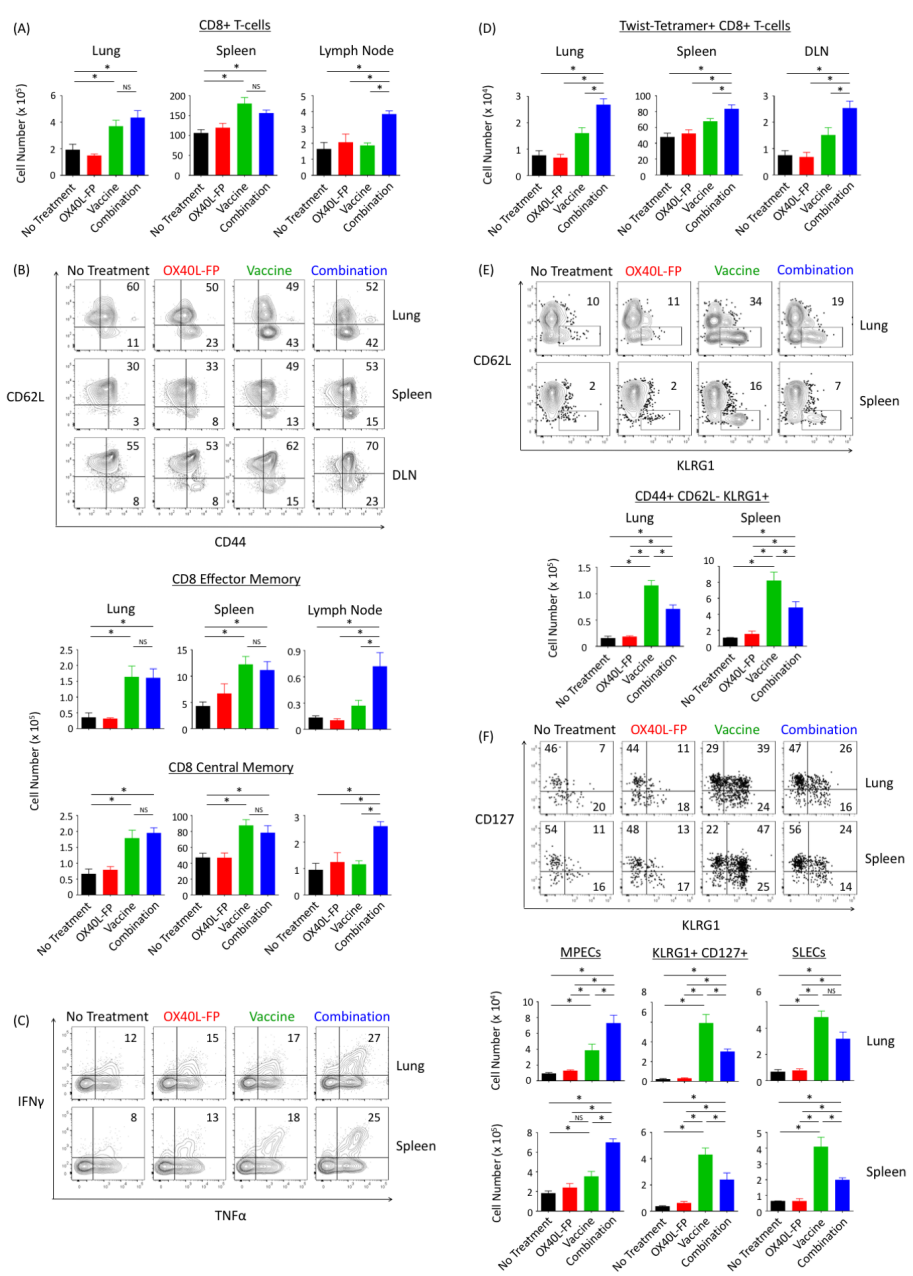
Combination therapy does not augment the total CD8^+^ Tem and Tcm populations in the lung, although it increases cytokine production, Twist-specific responses, and the formation of memory precursors **A.**, Absolute numbers of CD8^+^ T-cells in the lungs, spleens, and DLN (*n* = 5) on day 28 post tumor transplant. **B.**, Representative contour plots (denoting frequencies) of the CD8^+^ Tem (CD44^+^CD62L^-^) and Tcm (CD44^+^CD62L^+^) populations. **C.**, Representative contour plots of IFNγ and TNFα cytokine expression after anti-CD3/CD28 stimulation. Events were pre-gated on activated CD3^+^CD8^+^CD44^+^ populations. **D.**, Absolute numbers of Twist-tetramer^+^ CD8^+^ T-cells in lungs, spleens, and DLNs. **E.**, Representative contour plots and absolute numbers of activated CD3^+^CD8^+^CD44^+^ T-cells from lung and spleen expressing the terminal differentiating marker KLRG1. **F.**, Representative dot plots and absolute numbers of SLECs (KLRG1^+^CD127^-^) and MPECs (KLRG1^-^CD127^+^). Events were pre-gated on CD3^+^CD8^+^CD44^+^CD62L^-^ Tem populations.

Although the combination therapy augmented the pool of CD4^+^ T-cells in 4T1 tumor-bearing mice, it did not demonstrate any proliferative effects on CD8^+^ T-cell populations located in the lung and spleen. Compared to the untreated and OX40L-FP monotherapy cohorts, the number of CD8^+^ T-cells located in the lung and spleen were 2-fold and 1.8-fold greater, respectively, upon treatment with MVA-Twist-TRICOM alone. However, adding OX40L-FP to the vaccine did not further expand this population in either tissue compartment (Figure 6A). Similar trends were also observed in both the CD8^+^ Tem and Tcm subpopulations (Figure 6B). Nevertheless, the combination therapy enhanced CD8^+^ T-cell functionality, as demonstrated by an increase in the population of active cytokine producers. Intracellular cytokine staining revealed that combining the two immunotherapies increased the frequency of T-cells in the lung and spleen that were double-positive for the Th1 cytokines IFNγ and TNFα after anti-CD3/CD28 stimulation *ex vivo* (Figure 6C).

Despite the inability of OX40L-FP to expand polyclonal CD8^+^ T-cell populations generated by MVA-Twist-TRICOM in the lung and spleen, significant combination effects were observed in the vaccine DLN. Here, the combination therapy enriched the total CD8^+^ T-cell population by 1.8-fold compared to the individual treatments (Figure 6A), suggesting a possible enhancement of CD8^+^ T-cell priming. The combination also significantly increased the absolute number of CD8^+^ Tem and Tcm cells by 2.6-fold and 2.1-fold, respectively (Figure 6B).

Furthermore, the combination therapy significantly increased the CD8^+^ T-cell populations specific for tumor antigen Twist, compared to the untreated and monotherapy controls. This was demonstrated by a significant increase in the number of T-cells that were positively stained with Twist-tetramer in the lung, spleen, and DLN (Figure 6D). The expression of L-selection, as determined by CD62L MFI, was significantly lower (2.3-fold) on Twist-tetramer^+^ CD8^+^ T-cells from the combination group, suggesting that the Twist-specific immune population was skewed towards an effector memory phenotype (Supplementary Figure 2).

### OX40L-FP reduces terminally differentiated CD8^+^ T-cells and promotes the development of memory precursors in combination with MVA-Twist-TRICOM

Although no combination effects were observed in CD8^+^ T-cell populations of the lung or spleen (Figure 6A), OX40L-FP was able to promote the long-term memory potential of CD8^+^ Tem cells generated upon vaccination (Figures 6E and 6F). Compared to the untreated and OX40L-FP monotherapy controls, MVA-Twist-TRICOM increased the population of terminally differentiated KLRG1^+^ T-cells within the CD8^+^ Tem compartments of both the lung and spleen (Figure 6E). However, when combined with OX40L-FP, the total number of KLRG1^+^ CD8^+^ Tem cells significantly decreased 1.6-fold and 1.7-fold, respectively, although they were still more abundant than those in the untreated and OX40L-FP monotherapy groups (Figure 6E). This phenotypic change occurred regardless of CD127 expression, as the short-lived effector cell (SLECs; KLRG1^+^CD127^-^) and KLRG1^+^CD127^+^ subsets also increased with vaccination, and then subsequently declined in combination with OX40L-FP (Figure 6F). The reduction in KLRG1^+^ T-cell populations coincided with a corresponding increase in the population of memory precursor effector cells (MPECs; KLRG1^-^CD127^+^). The total number of MPECs in the lung and spleen were 2-fold greater (*P* < 0.05) in the combination group than in the MVA-Twist-TRICOM monotherapy group, suggesting that the addition of OX40L-FP expanded the pool of CD8^+^ Tem cells with the potential to develop into a long-lived, self-renewing memory population.

### Combination therapy abrogates the suppressive activity of Tregs

**Figure 7:**
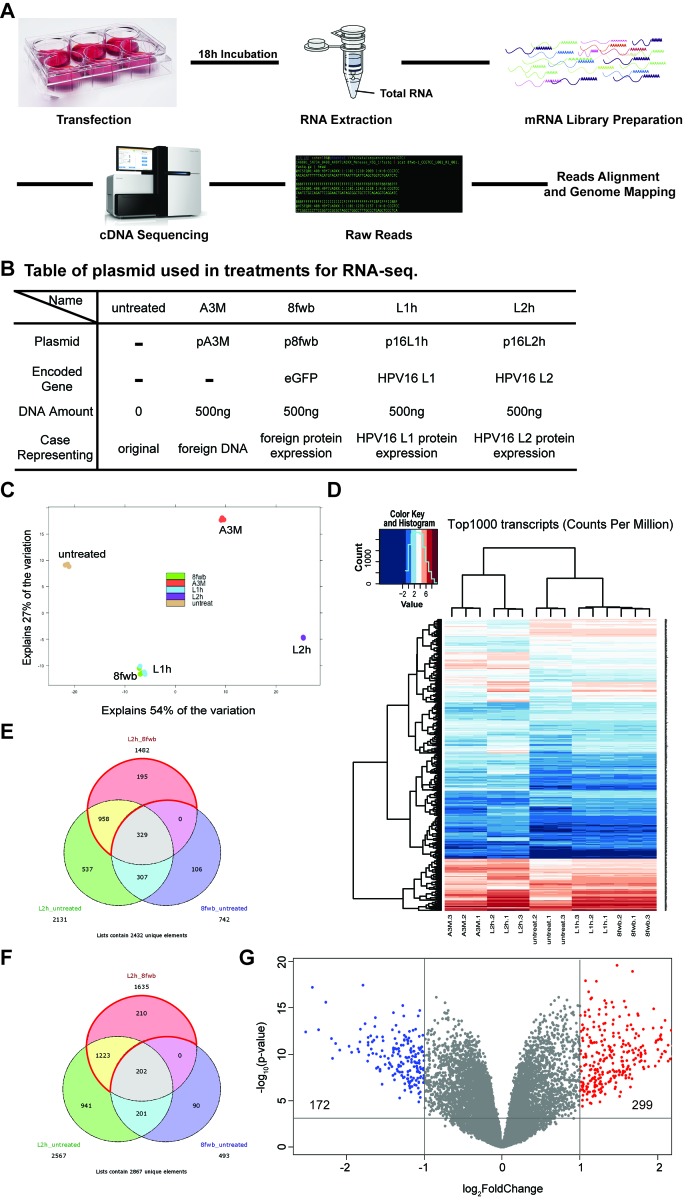
Combination therapy does not affect Treg numbers in the 4T1 tumor model; however, it reduces their suppressive activity **A.**, Absolute Treg numbers in lungs, spleens, and DLN (*n* = 5) on day 28 post tumor transplant. **B.**, CD4^+^ Teff:Treg and CD8^+^ Teff:Treg ratios in the lung. **C**., Treg suppression assay. Tregs pooled from lungs and spleens were co-cultured with CD4^+^ responder cells isolated from WT Balb/c splenocytes. CD4^+^ responders were stimulated with soluble anti-CD3 in the presence of irradiated APCs. H^3^-thymidine uptake was measured 4 days later. Background proliferation of Tregs was analyzed separately for each treatment.

Flow cytometric analysis demonstrated that, compared to the individual therapies, the combination of OX40L-FP and vaccine did not induce any significant changes in absolute Treg numbers located in the lung, spleen, or DLN. While MVA-Twist-TRICOM increased the Treg population 1.6-fold in the lung compared to the untreated control, combining both immunotherapies did not further enhance Treg numbers (Figure 7A). Despite the increase in Tregs following combination therapy, the CD4^+^ Teff:Treg ratio was 2.2-fold greater than that of the control treatments (*P* < 0.05). However, the CD8^+^ Teff:Treg ratio was no different in relation to the MVA-Twist-TRICOM group (Figure 7B).

*Ex-vivo* suppression assays demonstrated the ability of combination therapy to inhibit Treg functionality at the metastatic tumor site and in the periphery (Figure 7C). Tregs isolated on day 28 from both the lung and spleen of untreated mice impaired anti-CD3 induced proliferation of a common naïve CD4^+^ T-cell responder population by 40% and 45%, respectively. While the OX40L-FP and MVA-Twist-TRICOM monotherapies did not alter the inhibitory activity of Tregs, the combination therapy completely abrogated their suppressive capabilities, as they failed to modulate expansion of the CD4^+^ responder population, relative to its baseline proliferation in the absence of Tregs.

## DISCUSSION

OX40 agonists have been explored in combination with vaccine-based immunotherapies due to their ability to enhance tumor-reactive T-cell responses [5, 20]. Most notably, these costimulatory agents have been combined with tumor cell lysate and tumor-associated peptide vaccine platforms supplemented with anti-CD40 mAbs and TLR ligands that enhance antigen presentation by DCs [21-23]. OX40 agonists have also been combined with DC vaccines pulsed with soluble protein or apoptotic tumor cells [24], and with whole tumor cell vaccines secreting granulocyte-macrophage colony-stimulating factor (GM-CSF) [13, 25]. Other studies incorporated DC-targeted vaccines that facilitate cross-presentation of tumor antigens, such as the anti-DEC205/HER2 fusion antibody [26], and the gp96-Ig chaperone-secreting *IMPACT* cell-based vaccine [27].

We report here on the first study that effectively employed an antigen-specific recombinant viral vector (MVA-Twist-TRICOM) in combination with an OX40L fusion protein agonist (OX40L-FP) for cancer therapy. This combination therapy was evaluated for the treatment of pulmonary metastases overexpressing the epithelial-mesenchymal transition (EMT) transcription factor Twist in the 4T1 tumor model of TNBC. We previously investigated a different TRICOM-based viral construct that uniquely contained a transgene for OX40L [28], although delivering an OX40 agonist separate from the vaccine itself provides the flexibility to alter the timing and dose at which to initiate additional co-stimulatory signals. OX40L-FP selectively enhanced the proliferation of CD4^+^Foxp3^-^ T-cells *in vitro*, while having no direct co-stimulatory effect on the CD8^+^ population (Figure 1A). However, OX40L-FP can still indirectly augment CD8^+^ T-cell responses *in vivo* by enhancing CD4^+^ T-cell help, resulting in the elevated production of effector cytokines and the maturation of immunogenic APCs [29].

In the 4T1 tumor model, a significant reduction in lung metastasis, and a corresponding increase in overall survival, was observed only when OX40L-FP and MVA-Twist-TRICOM were administered together (Figure 3B and 3C). No effect on primary tumor growth was observed (Figure 3B, inset), possibly due to its low Twist expression. We previously reported [19] that the expression of Twist in biopsies of 4T1 pulmonary metastases was more than 4 times greater *vs*. primary tumors by RT-PCR, which is consistent with its role in promoting epithelial-mesenchymal transition. This finding was further confirmed by immunohistochemistry where the expression of Twist in pulmonary metastases was significantly elevated compared to primary tumors (*P* = 0.0373). This suggests that a vaccine regimen targeting Twist may be more effective in the treatment of metastatic lesions. Interestingly, although the combination therapy led to a significant reduction of metastatic lung deposits (Figure 3B), no mice had complete resolution of metastases. The reduction of metastases led to an increased survival advantage (Figure 3C), without a right sided survival ‘tail’. Considering the last treatment was administered on day 21 post tumor transplant, it is possible that additional vaccinations and OX40L-FP co-stimulatory boosts may further improve relapse of metastatic lesions in the lung. Depletion studies demonstrated that both CD4^+^ and CD8^+^ T-cell populations played critical roles in mediating anti-tumor activity (Figure 3D), which correlate with the findings of other combination studies that also demonstrate the engagement of both CD4^+^ and CD8^+^ T-cell immune responses to reduce tumor burden. 4T1 tumor cells do not express MHC class II. As the depetion studies indicate that both CD8 T-cells cells and to a lesser degree CD4 T-cells were needed for antitumor activity (Figure 3D), we hypothesize that the role of antigen-specific CD4 T-cells in the antitumor response is to provide cytokine support for the antigen-specific CD8 T-cells.

Immune subset analyses revealed that the combination therapy not only enhanced CD4^+^Foxp3^-^ T-cell expansion at the metastatic tumor site (lung) and in the periphery (spleen and DLN), but also augmented memory formation compared to the untreated and monotherapy controls. In particular, the combination therapy significantly increased the frequency of CD4 Tem cells, although no such change was observed in the CD4 Tcm subpopulation (Figure 4A and 4B). This latter result supports the work of Soroosh, et al. [30], who discovered that OX40 signaling imprints a survival program that selectively promotes the lineage commitment and expansion of the CD4^+^ Tem pool.

While the combination therapy enhanced CD4^+^Foxp3- T-cell responses, it did not increase total CD8^+^ and memory CD8^+^ T-cell populations in the lung or spleen. Although MVA-Twist-TRICOM alone increased the absolute number of total CD8^+^ T-cells, and the CD8^+^ Tem and Tcm subsets, adding OX40L-FP to the vaccine monotherapy did not further expand any of these populations. Despite having minimal effects in lung and splenic tissues, the combination therapy was able to increase CD8^+^ T-cell numbers in the vaccine DLN, suggesting a boost in CTL priming and homing to this lymphoid compartment (Figure 6A and 6B). OX40 signaling has shown the ability to induce greater T-cell migration into secondary lymphoid organs *via* up-regulation of chemokine receptors, such as CXCR5; however, such mechanisms were not studied here [3].

Although OX40L-FP did not augment the total CD8^+^ T-cell population in the lung, it alternatively skewed the long-term survival and memory potential of CD8^+^ Tem cells in combination with vaccine. MVA-Twist-TRICOM increased the population of terminally differentiated KLRG1^+^ T-cells, including both the CD127^+^ and CD127^-^ (SLECs) subsets (Figure 6E and 6F). However, adding OX40L-FP to the vaccine monotherapy shifted the CD8^+^ Tem population towards a memory phenotype, as demonstrated by a higher abundance of KLRG1^-^ MPECs that co-expressed CD127 (IL-7Rα), which is essential for homeostatic maintenance and proliferation of memory T-cells (Figure 6F) [31]. This result is supported by previous studies establishing that OX40 signals delivered during priming critically potentiate the maintenance, survival, and self-renewal capabilities of KLRG1^-^ memory CD8^+^ T-cells [32]. These memory cells not only produce IL-2, but their levels of IL-2 production are impaired in the absence of OX40 signals.

Combination therapy also enhanced the functionality of CD8^+^ T-cells in the lung, increasing the number of IFNγ and TNFα cytokine producers within the activated CD44^+^ population upon anti-CD3/CD28 stimulation, and suggesting that the combination therapy prevented the onset of T-cell exhaustion (Figure 6C). Despite enhanced cytokine production, the combination therapy did not affect the lytic potential of CD8^+^ T-cells. Degranulation of the activated CD8^+^ T-cell population, as determined by CD107a translocation following *ex vivo* stimulation, was unaffected by the combination therapy (data not shown). This result is in accordance with previous studies suggesting that OX40-OX40L signals do not necessarily augment CD8^+^ effector function, but are rather more essential for promoting T-cell survival, memory formation, and rapid recall responses [29]. However, others have reported the opposite, whereby OX40 ligation can additionally increase granzyme B expression, cytotoxicity, and protective capacity on a per-cell basis [33].

One of the most important features of the combination therapy was its ability to enhance both CD4^+^ and CD8^+^ T-cell responses against the tumor antigen Twist in the lung (Figures 4C and 6D), which likely resulted from increased T-cell priming at the vaccine DLN (Figures 4B and 6B). Increasing the population of Twist-specific T-cells is essential for directing the immune response towards distant micro- and macro-metastatic 4T1 lesions in the lung. Enhanced 4T1 tumor targeting was visualized by IHC analysis of lung tissue sections, whereby regions of metastasis, characterized by elevated Twist expression, had significantly greater levels of CD4^+^ T-cell infiltrate after the combination treatment (Figure 5C).

The anti-tumor and immunomodulatory activity of the combination therapy may also be potentiated by the inhibitory effects of OX40L-FP on Tregs [34, 35]. Previous reports have suggested that OX40 ligation can either augment or prevent Treg expansion depending upon the cytokine milieu during stimulation [36]. However, we show here that OX40L-FP did not increase Treg populations in the 4T1 mouse model, either alone or in combination with MVA-Twist-TRICOM (Figure 7A). Instead, OX40L-FP had a significant effect on Treg functionality when administered together with vaccine, as Tregs isolated from the lungs and spleens of mice in the combination groups were less immunosuppressive than those from the monotherapy control groups (Figure 7B). These effects were observed on day 28 post tumor transplant, or 8 days after the last OX40L-FP injection, which is a surprising observation considering the half-life of OX40L-FP in mouse serum is less than a day (data not shown). As a result, the combination therapy is able to reduce the immunosuppressive barriers of the tumor microenvironment, and overcome peripheral tolerance mechanisms mediated by Tregs, for an extended period of time, maximizing T-cell effector function.

This study demonstrates that combining OX40L-FP and MVA-Twist-TRICOM vaccine impairs tumor metastasis by inducing T-cell expansion and memory formation, and that such effects are likely facilitated by inhibiting Treg-mediated suppression. Furthermore, combining OX40 agonists and cancer vaccines can generate effective anti-tumor immune responses against nuclear proteins that are difficult to target with small molecule inhibitors or monoclonal antibodies. Ultimately, this strategy can be applicable to other “undruggable” transcriptional factor targets presented on the surface of tumor cells in the form of peptide-loaded MHC class I/II complexes, including brachyury, a driver of EMT in TNBC demonstrating considerable promise as a clinical target of cancer vaccines [37-39].

## MATERIALS AND METHODS

### Tumor model

4T1 murine breast carcinoma cells were purchased from American Type Culture Collection, cultured in the recommended media, and implanted (5x10^4^ cells) into the mammary fat pad of 10-14-week old wild-type Balb/c mice obtained from the NCI Frederick Cancer Research Facility. Tumor-inoculated mice were subsequently treated with OX40L-FP (10 mg/kg, IP) and MVA-Twist-TRICOM (1x10^8^ PFU, sc), which were obtained from MedImmune LLC and Bavarian Nordic, respectively, under separate Cooperative Research and Development Agreements (CRADAs). MVA-Twist-TRICOM was administered on days 7, 14, and 21 after tumor implant, while OX40-FP was administered on days 10, 13, 17, and 20. Mice were sacrificed on day 28 when primary tumor sizes from untreated mice reached their ethical limits (2000 mm^3^). Pulmonary metastases were evaluated by enumerating the number of clonogenic 4T1 tumor colonies cultured from each lung, as previously described [18].

### Survival study with CD4^+^ /CD8^+^ T-cell depletion

In separate studies for survival, 4T1 primary tumors were surgically removed on day 15 after implantation. T-cells were depleted using a 100 µg dose of either anti-CD4 (Clone GK1.5) or anti-CD8 (Clone 2.43) (BioXcell) administered intraperitoneally the first 3 days following tumor implantation, and then once per week thereafter, beginning on day 7.

### Flow cytometry analysis

T-cell subsets in lung, spleen, and vaccine DLN were analyzed on a FACSVerse flow cytometer (BD Biosciences). Here, DLN refers to lymph nodes draining the site of vaccination (the MVA-Twist-TRICOM vaccine was administered subcutaneously into the flank). Lungs were digested in an RPMI cocktail composed of 2 mg/mL collagenase type I and 40 U/mL DNase I. Lymphocytes were then enriched *via* 40%/70% percoll gradient centrifugation. Cells were permeabilized with Foxp3/Transcription Factor Staining Buffer Set (eBioscience). Live/Dead Fixable Yellow Dead Cell Stain Kit (Thermo Fisher Scientific) was utilized to exclude dead cells during analysis. The following antibodies from BD Biosciences and eBioscience were used for staining: CD8-FITC (53.67), Foxp3-PE (FJK-16s), CD3-AF700 (17A2), CD44-PerCP-Cy5.5 (IM7), CD4-FITC (RM4-5), CD62L-BV421 (MEL-14), and CD25-APC (PC61). MHC class I–restricted (H2-K^d^) PE-labeled Twist-tetramer (sequence: LYQVLQSDEL) was synthesized in-house at the NCI Core Facility.

Th1 cytokine production was evaluated *via* intracellular cytokine staining. Whole splenocytes were stimulated for 6 hours at 37ᴼC with 1 µg/mL soluble anti-CD3 (145-2C11) and 2 µg/mL soluble anti-CD28 (37.51) in the presence of Golgi Stop and Plug (BD Biosciences). Lymphocytes from lungs were stimulated with soluble anti-CD3/CD28 on ice for 20 minutes, followed by cross-linking with goat-anti-hamster IgG (SouthernBiotech). Cells were stained with IFNγ-BV421 (XMG1.2) and TNFα-APC (MP6-XT22) (BD Biosciences).

### CD4^+^ T-cell and Treg functional assays

Lymphocyte proliferation assays were conducted as previously described to characterize CD4^+^ Twist-specific responses following immunotherapy [18]. T-cells were stimulated with 2.5 µg/mL of MHC class II–restricted (H2-IE^d^) Twist peptide (sequence: QQPASGKRGARKRRS, NCI Core Facility). Treg suppression assays were also conducted as previously described [40]. Briefly, Tregs were isolated from each treatment group and co-cultured with a common syngeneic CD4^+^ T-cell responder population purified from splenocytes of naïve mice.

### Immunohistochemistry

Lung tissue was frozen in OCT compound and sectioned for IHC staining. Sections were fixed in methanol, permeabilized with 0.5% saponin, and blocked with 10% normal goat serum /0.1% Tween-20 (Thermo Fisher). They were incubated with primary antibody overnight at 4ᴼC, HRP-labeled secondary antibodies for 1 hour at RT, and then DAB substrate (Vector Laboratories), followed by hematoxylin counterstaining. The following antibodies were used: rat-anti-mouse CD4 (GK1.5; BioRad), rabbit-anti-mouse Twist1 (polyclonal; EMD Millipore), and goat-anti-rabbit and goat-anti-rat IgG (Thermo Fisher). Images were acquired with Aperio ImageScope (Leica Biosystems).

### Statistical analysis

Each experiment was repeated at least once and the data shown are representative of the results that were attained each time. Statistical analyses were performed using 2-tailed Student’s *t*-tests with 95% confidence. Probability values of *P* < 0.05 were considered significant and denoted with an asterisk in figures. Survival analysis was performed with the log-rank test. All bar graphs report mean ± SEM.

## SUPPLEMENTARY MATERIALS FIGURE



## Abbreviations

AF: Alexa fluor
APCs: Antigen presenting cells
CD: Cluster of differentiation
CRADA: Cooperative Research and Development Agreements
CTL: Cytotoxic T lymphocyte
DAB: 3,3’-diaminobenzidine
DCs: Dendritic cells
DLN: Draining lymph node
EMT: Epithelial-mesenchymal transition
FBS: Fetal bovine serum
FITC: Fluorescein isothiocyanate
GM-CSF: Granulocyte macrophage colony stimulating factor
HER2: Human epidermal growth factor receptor two
IFNγ: Interferon gamma
IHC: Immunohistochemistry
IP: Intraperitoneal
KLRG1: Killer cell lectin-like receptor subfamily G member 1
MFI: Mean fluorescence intensity
MHC: Major histocompatibility complex
MPEC: Memory Precursor effector cell
MVA: Modified vaccinia ankara
OCT: Optimum cutting temperature
OX40-FP: OX40 Fusion Protein
PE: Phycoerythrin
PerCP: Peridinin Chlorophyll Protein Complex
RT: room temperature
SC: Subcutaneous
SLEC: Short-lived effector cell
Tcm: Terminal central memory
Teff: T-effector cell
Tem: Terminal effector memory
Th1: Type 1 T helper
TNBC: Triple negative breast cancer
TNFR: Tumor necrosis factor receptor
TNFα: Tumor necrosis factor alpha
Tregs: Regulatory T-cells
TRICOM: Triad of Costimulatory Molecules (B7-1, ICAM-1, LFA-3)
WT: Wild-type
