# Supplementary material for: Combination therapy with an OX40L fusion protein and a vaccine targeting the transcription factor twist inhibits metastasis in a murine model of breast cancer
Source: Oncotarget. 2017 Aug 5;8(53):90825–41. doi: 10.18632/oncotarget.19967 (PMC5710887; doi:10.18632/oncotarget.19967)
Supplement: Supplementary file 1 [file oncotarget-08-90825-s001.pdf]

Combination therapy with an OX40L fusion protein and a vaccine targeting the transcription factor twist inhibits metastasis in a murine model of breast cancer

SUPPLEMENTARY MATERIALS

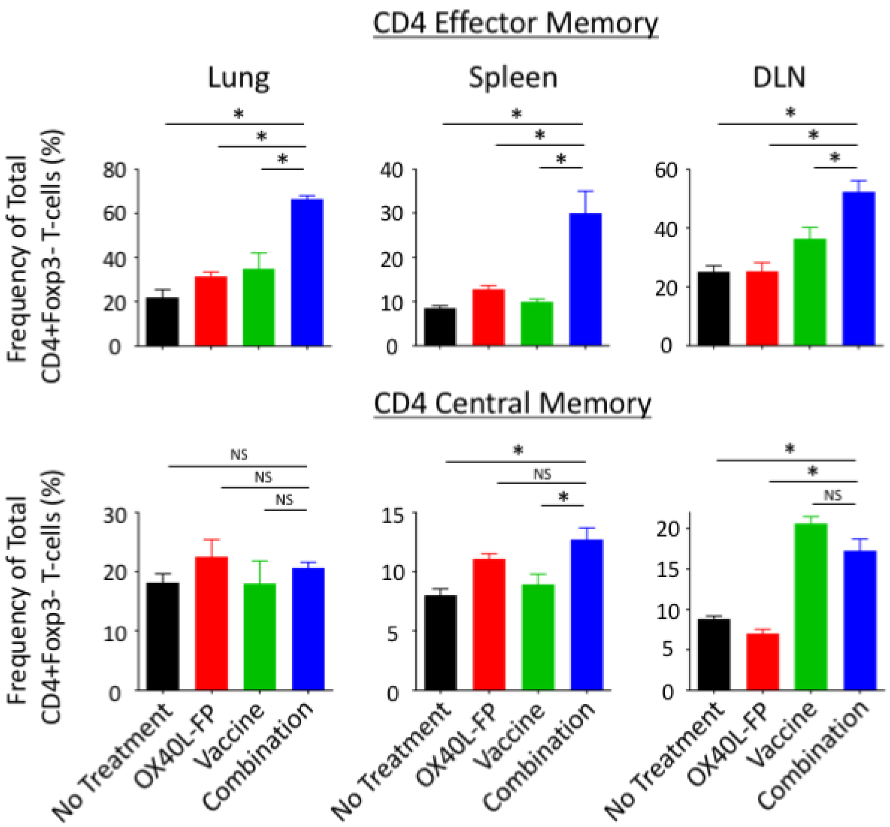

Supplementary Figure 1: Combination therapy increases the frequency of CD4+ Tem cells in 4T1 tumor-bearing mice, but it does not affect the frequency of CD4$^+$ Tcm cells. Frequency of CD4$^+$ Tem (CD44$^+$CD62L$^-$) and Tcm (CD44$^+$CD62L$^+$) populations in the lungs, spleens, and DLNs (n=5) on day 28 post tumor transplant. Events were pre-gated on CD3$^+$CD4$^+$Foxp3$^-$ T-cells.

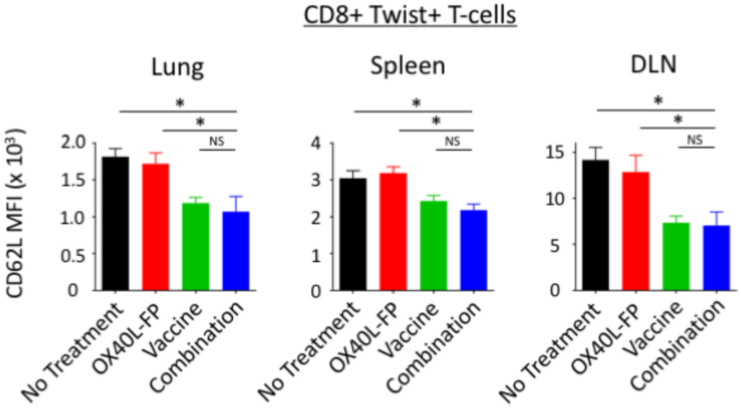

Supplementary Figure 2: Combination therapy reduces the surface expression of L-selectin (CD62L) on CD8$^+$ Twist-Tetramer$^+$ T-cells. MFI (Mean fluorescence intensity) of CD62L on Twist-specific CD8$^+$ T-cells in the lungs, spleens, and DLNs (n=5) on day 28 post tumor transplant. Events were pre-gated on CD3$^+$CD8$^+$Twist-tetramer$^+$ T-cells.
